# Protective Immunity Against Hepatitis C: Many Shades of Gray

**DOI:** 10.3389/fimmu.2014.00274

**Published:** 2014-06-16

**Authors:** Mohamed S. Abdel-Hakeem, Naglaa H. Shoukry

**Affiliations:** ^1^Centre de Recherche du Centre Hospitalier de l’Université de Montréal (CRCHUM), Montréal, QC, Canada; ^2^Département de Microbiologie, Infectiologie et Immunologie, Faculté de Médecine, Université de Montréal, Montréal, QC, Canada; ^3^Department of Microbiology and Immunology, Faculty of Pharmacy, Cairo University, Cairo, Egypt; ^4^Département de Médecine, Faculté de Médecine, Université de Montréal, Montréal, QC, Canada

**Keywords:** hepatitis C, acute infection, HCV reinfection, innate immunity, adaptive immunity, NK cells, T cells, antibodies

## Abstract

The majority of individuals who become acutely infected with hepatitis C virus (HCV) develop chronic infection and suffer from progressive liver damage while approximately 25% are able to eliminate the virus spontaneously. Despite the recent introduction of new direct-acting antivirals, there is still no vaccine for HCV. As a result, new infections and reinfections will remain a problem in developing countries and among high risk populations like injection drug users who have limited access to treatment and who continue to be exposed to the virus. The outcome of acute HCV is determined by the interplay between the host genetics, the virus, and the virus-specific immune response. Studies in humans and chimpanzees have demonstrated the essential role of HCV-specific CD4 and CD8 T cell responses in protection against viral persistence. Recent data suggest that antibody responses play a more important role than what was previously thought. Individuals who spontaneously resolve acute HCV infection develop long-lived memory T cells and are less likely to become persistently infected upon reexposure. New studies examining high risk cohorts are identifying correlates of protection during real life exposures and reinfections. In this review, we discuss correlates of protective immunity during acute HCV and upon reexposure. We draw parallels between HCV and the current knowledge about protective memory in other models of chronic viral infections. Finally, we discuss some of the yet unresolved questions about key correlates of protection and their relevance for vaccine development against HCV.

## Introduction

Hepatitis C virus (HCV), a member of the family *Flaviviridae*, is a non-cytopathic hepatotropic virus transmitted primarily through contaminated blood (1). The World Health Organization (WHO) estimates that there are 185 million individuals infected with HCV worldwide (2, 3). The prevalence is higher in developing countries, the highest being in Egypt where it is estimated that 15–20% are seropositive for HCV (4, 5). These numbers are probably an underestimate, since many HCV-infected individuals have not been tested and thus remain undiagnosed.

The acute phase of hepatitis C is empirically defined as the first 6 months following infection. Approximately, 20–30% of infected individuals are able to clear the virus spontaneously without any therapeutic intervention during this phase while 70–80% become persistently infected (1). As the virus continues to replicate in the liver of chronically infected individuals, they develop a variety of liver diseases over a period of 5–30 years including hepatic fibrosis, end-stage cirrhosis, and hepatocellular carcinoma (HCC) (6). Such patients represent 25% of the cirrhosis and HCC cases worldwide (7), making hepatitis C the most common indication for liver transplantation in North America (8, 9).

Despite the progress in understanding immunity against HCV infection and its pathogenesis and, the development of highly effective direct-acting antivirals (DAAs), a prophylactic anti-HCV vaccine is still lacking. In absence of such a vaccine, two million new HCV infections are estimated to occur every year (10). Such cases are highly prevalent among marginalized populations like injection drug users (IDUs), men who have sex with men (MSM), and individuals living in developing countries with limited access to screening and treatment (11, 12). Vaccine development against HCV is hampered by our limited knowledge of what constitutes an effective and protective immune response against HCV, as well as protection in real life exposure settings. Many shades of gray remain and constitute active areas of the current research on hepatitis C. In the following pages, we will attempt to review the correlates of protection against HCV and identify these outstanding questions and the ongoing efforts to address them.

## The Virus

**Figure 1:**
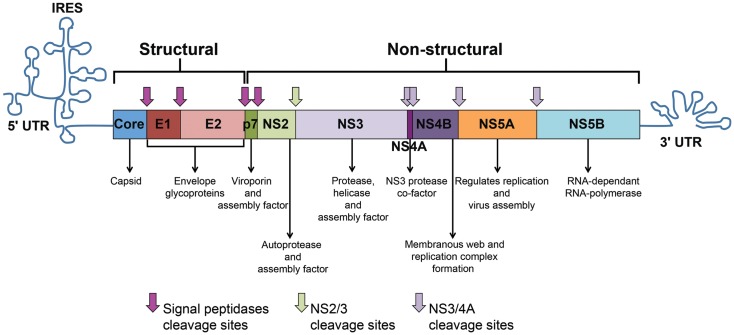
**HCV genome and polyprotein**. The HCV genome is composed of an open reading frame (ORF) flanked by 5’ and 3’ untranslated regions (UTRs). IRES-mediated translation of the ORF leads to the formation of a polyprotein that is processed into 10 viral proteins. Cleavage of the core protein from E1 involves cellular signal peptidases, which also cleave E1, E2, and p7 from the polyprotein (pink arrows). The NS2–NS3 protease auto-cleaves itself (green arrow). The NS3 protease located in the first one-third of NS3, assisted by its membrane-bound cofactor, NS4A, cleaves the remaining proteins NS3, NS4A, NS4B, NS5A, and NS5B (violet arrows).

The HCV genome consists of an uncapped positive single-stranded RNA (+ssRNA) of approximately 9.6 kb-pairs (13). The genome represents an uninterrupted open reading frame (ORF) encoding a polyprotein precursor of approximately 3000 amino acids (13). The viral genome includes 5^′^ and 3^′^ untranslated regions (UTRs) that contain secondary RNA structures essential for viral replication. The 5^′^ UTR harbors an internal ribosome entry site (IRES) where protein translation is initiated. The HCV polyprotein is processed co- and post-translationally by a combination of cellular and viral proteases into three structural proteins (Core, E1, and E2) and seven non-structural (NS) proteins (P7, NS2, NS3, NS4A, NS4B, NS5A, and NS5B) (Figure 1). Core is the building unit of the viral nucleocapsid. The envelope glycoproteins E1 and E2 interact with the viral receptors on permissive cells and mediate viral entry. E2 also contains hypervariable regions (HVR) that are targeted by neutralizing antibodies (nAbs). P7 acts as a viroporin or ion channel. NS2 possesses an autoprotease activity necessary for the polyprotein cleavage between NS2 and NS3. NS3 acts as serine protease in combination with NS4A that acts as a cofactor to catalyze the processing of the HCV polyprotein. NS3 also harbors RNA helicase/NTPase activity that unwinds RNA–RNA substrates and is essential for viral replication [reviewed in Ref. (14, 15)]. The functions of NS4B and NS5A are poorly characterized. However, studies show that NS4B induces the formation of a membranous web compartment where viral replication takes place (16), and cell-culture adaptive mutations mapped to the NS5A enhance RNA replication suggesting its importance for viral replication (17, 18). NS5A was also shown to harbor a region that may determine response to alpha interferon (IFN-α) therapy known as interferon sensitivity determining region (ISDR) (19). NS5B is the viral RNA-dependent RNA-polymerase (RdRp) responsible for HCV–RNA replication (14). As with other RNA viruses, the HCV RdRp enzyme lacks proof-reading activity and is highly error prone, leading to the emergence of different viral populations circulating in the blood of an individual patient as a mosaic of highly related sequences termed “quasispecies” (14).

Hepatitis C virus is classified into seven genotypes (1–7) and up to 67 different subtypes based on the nucleotide sequences of the Core/E1 and NS5B regions (20). The different genotypes show marked differences in geographic distribution, response to therapy, and pathogenesis (15).

## Viral Replication and Life Cycle

Hepatitis C virus replicates primarily in human hepatocytes. The first described receptors for HCV were CD81 (21) and scavenger receptor class B type I (SR-BI) (22). The LDL receptor and glycosaminoglycans (GAG) mediate initial binding to susceptible cells prior to interaction with CD81 and SR-BI (15). Tight junction proteins claudin-1 (CLDN1) and occludin (OCLN) act as receptors/co-receptors for HCV at cell junctions (23, 24). It was recently reported that the cholesterol absorption receptor Niemann–Pick C1-like 1 (NPC1L1) is an essential entry factor (25) and that the epidermal growth factor receptor (EGFR) and ephrin receptor type A2 are also required for HCV entry and possibly modulate the interaction between CD81 and CLDN1 (26). Human CD81 and OCLN are the minimally required receptors to render mouse hepatocytes susceptible to HCV entry (24). Binding of HCV to its receptors is followed by fusion of the viral envelope with the cellular membrane by clathrin-mediated endocytosis then fusion with the endosomal membrane and the viral genome is released into the cytosol (27). Cap-independent IRES-mediated translation of the HCV ORF generates a large polyprotein that is subsequently processed into mature structural and NS proteins (Figure 1). Junctions between structural proteins are processed by host signal peptidases from the endoplasmic reticulum. The viral NS proteins are processed by the NS2/3 autoprotease (14) and the NS3/4A serine protease (28). Replication takes place within ER derived structures known as the membranous web and is mediated by NS5B through a negative strand intermediate in a replication complex. Viral assembly occurs within lipid raft like structures. HCV nucleocapsid is built from units of the core protein with RNA, surrounded by a membrane derived from the human cell with embedded heterodimers of the envelope glycoproteins E1 and E2 [reviewed in Ref. (15)]. The virions associate with low-density and very-low-density lipoproteins (LDL and VLDL) forming lipoviroparticles (LVPs) that are pleomorphic (29).

## Models for Studying HCV Infection and Immunity

### *In vitro* models

Hepatitis C virus replicates poorly in tissue culture. Earlier surrogate models to study HCV protein functions, virus–host interaction, and viral entry included vaccinia virus (VV) vectors expressing HCV proteins, direct transfection of HCV RNA, subgenomic, and full length replicons and viral pseudoparticles carrying HCV envelop glycoproteins on a capsid backbone of vesicular stomatitis virus or lentiviruses (HCVpp). It was not until 2005 that the first *in vitro* replicating strain was isolated from a Japanese patient with fulminant hepatitis termed JFH-1 virus, a genotype 2a isolate (30–32). Even with the development of this system, very few cell lines are permissive for its replication, often involving adaptive mutations within the viral genome and/or impairment in some of the cellular antiviral mechanisms [reviewed in Ref. (15, 33)]. These models have been instrumental in studying the innate antiviral response against HCV on a cellular level and identification of many of the underlying viral evasion mechanisms. The development of new cell lines or methods that allow HCV replication in primary human or mouse hepatocytes is an area of intense research.

### *In vivo* models

Humans and chimpanzees are the only two species that are susceptible to HCV infection. The chimpanzee model has been instrumental in the early studies of immunity against HCV where timing of the infection and infecting viral strains were known and it was possible to examine intrahepatic immune responses. Research on chimpanzees is now restricted (34) and the search for an alternate animal model is ongoing. Although considerable progress has occurred in developing humanized mice susceptible to HCV infection, these mice are generated on immune deficient backgrounds that preclude studying adaptive immune responses. Cotransplantation of human CD34^+^ human hematopoietic stem cells and hepatocyte progenitors in mice with inducible liver damage demonstrated good engraftment of human leukocytes and hepatocytes. These mice became infected with HCV and demonstrated some HCV-specific immune responses and liver fibrosis (35). These data are preliminary and the model remains technically challenging. It will likely be a few more years before we have a suitable alternative to the chimpanzee model for studying HCV-specific immunity and preclinical testing of vaccine candidates [reviewed in Ref. (36)].

Due to the asymptomatic nature of HCV, a limited number of individuals present to the clinic with acute symptomatic infection. In that situation, it is usually difficult to determine the exact date of infection or exposure and the infecting viral strain(s). Most of our early knowledge about acute HCV came from studies of experimental infection of chimpanzees, or individuals infected following high risk exposures like needle stick injuries in health care workers, blood transfusions, as well as the few cases presenting with symptomatic acute HCV. Recent studies relied upon monitoring high risk individuals, in particular IDUs who currently represent the main population of novel HCV infection in developed countries. It is noteworthy that in these situations the definition of acute HCV can vary from one cohort to another and is dependent on the follow-up interval, where the date of infection is estimated at best. It is also ethically impossible to obtain liver biopsies during acute infection and our knowledge of acute intrahepatic responses is derived from the chimpanzee model.

## Clinical Course of HCV Infection

Hepatitis C virus RNA can be detected in the peripheral blood of infected individuals within one week following infection. Despite this high level of viral replication, HCV-specific immune responses remain undetectable in most infected individuals for several weeks suggesting that the virus outpaces the immune system and impairs its responses (37). Nevertheless, interferon stimulated genes (ISGs) are detected early in the liver and some mediators of innate immunity like natural killer (NK) cells are activated and can be detected in peripheral blood (discussed below). At approximately 4–8 weeks, HCV-specific CD4 and CD8 T cells become detectable in the liver and peripheral blood and are often but not always associated with an increase in liver transaminases in the blood (38, 39). Most individuals who are able to spontaneously clear HCV do so within the first 6 months and a smaller proportion may clear their infection within 12 months. The outcome of HCV infection is determined by a “ménage-à-trois” between host genetics, the virus and HCV-specific immunity. Other host factors that influence the outcome of infection include ethnicity, female sex, and accompanying co-morbidities (e.g., co-infection with human immunodeficiency virus (HIV), alcohol abuse, renal disease, obesity, or liver fibrosis) (40).

## Genetic Factors That Influence the Outcome of Acute Primary HCV

Several genetic factors, mostly in genes related to innate and adaptive immune functions, influence the outcome of acute HCV infection. Individuals homozygous for the killer inhibitory receptor (KIR) gene KIR2DL3 and its ligand HLA-C1 alleles are more likely to clear HCV infection than individuals with other KIR2DL:HLA-C combinations (41). KIRs interact with their cognate MHC class I ligands and regulate the activation and function of NK cells. KIR2DL3 has a lower affinity for HLA-C1 than other KIR2DL alleles, so HLA-C1-mediated inhibition of NK cells is thought to be weaker in individuals homozygous for KIR2DL3 and HLA-C1 thus enhancing their capacity to inhibit HCV replication or kill virally infected hepatocytes (42). The same KIR/HLA-C combination was consistently more frequent in IDUs with high risk exposure to HCV who remain seronegative/aviremic as compared to those with chronic infection (43).

Genome-wide association studies (GWAS) demonstrated a correlation between several single-nucleotide polymorphisms (SNPs) near the IL28B (IFNλ3) gene locus and the spontaneous resolution of infection, as well as response to IFN therapy (44–47). Although several SNPs were identified, the rs12979860 SNP has become the most relevant (48). The favorable allele was identified as CC and the non-favorable allele CT or TT for heterozygous or homozygous individuals, respectively. These alleles were found to be unequally distributed among individuals of different ethnicities (Asians, Caucasians, and African Americans) and correlated with their differential response to IFN therapy (44, 47). Recent data have reported a novel transiently induced region that carries a dinucleotide variant ss469415590 (TT or ΔG), which is in high linkage disequilibrium with rs12979860. This resulted in a frameshift variant that created a novel gene, designated IFNL4, encoding the interferon-λ4 protein (49). This mutation was also associated with higher levels of induction of IL28B and interferon gamma-induced protein 10 (IP-10) thus providing a potential mechanism for its enhanced role in viral clearance (50).

It is not yet clear how polymorphism in the IL28B and/or IFNL4 gene influences immune responses and the capacity to spontaneously eliminate HCV. Given that the IL28B and IL29 (IFNλ1) genes are in opposite orientation, this SNP is up stream of the two genes and may influence expression of both. The responder genotype was linked to higher expression of the IL28A/B as measured by qRT-PCR in PBMCs or protein levels in plasma and lower HCV viral load, but results were not consistent among different studies (45, 51, 52). IL-29 was upregulated during acute and chronic HCV and its expression correlated with induction of ISGs but not acute infection outcome (52, 53). The non-favorable IL28B genotype was associated with higher expression of ISGs in the liver (54–56), considered a predictor of non-response to IFN therapy. Interestingly, ISGs were differentially expressed in liver versus macrophages, where the non-responder genotype was associated with lower ISG expression in macrophages (56).

It was also suggested that IL28B polymorphism may influence NK cell function or the cross-talk between innate and adaptive immunity. Dring et al. have demonstrated that KIR2DS3 synergizes with IL28B to predict chronic evolution of HCV (57). Suppiah et al. have demonstrated that polymorphism in IL28B, HLA-C, and KIRs additively and interactively predict response to therapy in chronic HCV infection (58). However, functional data supporting this possibility are still lacking.

A more recent GWAS study demonstrated that in addition to IL28B, another SNP near the gene for the HLA class II molecule DQB1*03:01, was independently associated with spontaneous resolution of HCV infection (59). Indeed, several HLA HLA class I and class II alleles were previously associated with the outcome of HCV [reviewed in Ref. (60)]. The HLA class I alleles HLA-A*03, HLA-B*27, and HLA*B57 were associated with spontaneous clearance [reviewed in Ref. (60)]. Interestingly, the HLA-B*27 and -B*57 alleles are also associated with better viral control in HIV infected individuals (61). The HLA class II alleles HLA-DRβ1*0101, -DRB1*0401, -DRB1*1101, and DQB1*0301 were strongly associated with HCV clearance, whereas HLA-DRβ1*0701 was associated with HCV persistence (62–64). It is suggested that such HLA class I and II alleles favor the generation of immune responses targeting conserved epitopes or epitopes that are less likely to mutate because of the high fitness cost associated with such mutations and viral replication (65).

Other genetic factors that were associated with spontaneous clearance of HCV include: ISGs, toll-like receptors (TLRs), chemokines, cytokines, and their receptors [reviewed in Ref. (60)], as well as tapasin associated with peptide loading on MHC class I (66). Finally, HCV was also shown to exploit “holes” in the T cell repertoire to facilitate its escape from detection by virus-specific CD8 T cells (67).

## Innate Immune Responses during Acute Primary HCV

### Viral sensing and intracellular innate immunity

Foreign RNA molecules are recognized by pattern recognition receptors (PRRs) including TLRs and/or intracellular nucleic acid-binding proteins (68, 69). In hepatocytes, TLR3, protein-kinase R (PKR), and retinoic-acid-inducible gene I (RIG-I) are the main PRRs recognizing HCV upon entry and replication. PKR and RIG-I activation signals are relayed via the mitochondrial antiviral signaling protein (MAVS), while TLR3 signals are relayed via the TIR domain-containing adapter inducing IFN-β (TRIF) [reviewed in Ref. (70)]. Downstream of these adaptor proteins signaling cascades are activated culminating in the secretion of type I IFNs (70). Binding of auto- and paracrine type I IFNs to their receptors activates signaling along the JAK–STAT signaling pathway. This induces the expression of hundreds of ISGs in the infected cells and neighboring cells (71) and creates a general antiviral state in the liver that limits HCV RNA replication and cell-to-cell spread (72). ISGs expressed in response to HCV infection include several proteins with known antiviral effects, as well as some others that were shown to promote HCV replication *in vitro*, as ISG15 and USP18 (73, 74).

Upregulation of ISGs is detected in the liver early after HCV infection irrespective of the outcome, suggesting that most HCV isolates are resistant to the antiviral effects of this early innate response (75–78). Nevertheless, the pro- and anti-viral roles of the different ISGs would suggest differential induction of specific ISGs in patients with different outcomes of HCV infection. Recent work on primary hepatocytes infected with HCV demonstrated that type III IFNs (IFNλs) could be the earliest responders and that they lead to induction of ISGs shared with type I IFNs, as well as other distinct ISGs (79, 80). Preliminary studies suggest that although similar ISGs are induced by type I and type III IFNs, they may signal through two distinct pathways (81), have different kinetics (82) maybe cell type specific (56) and/or IFNλ allele dependent (83).

Infected hepatocytes are not the only source of type I and type III IFNs but liver resident Kupffer cells (84), BDCA3^+^ myeloid dendritic cells (mDCs) (85), and plasmacytoid DCs (pDCs) (86, 87) can detect HCV RNA and produce type I and type III IFNs, thus contributing to the intrahepatic antiviral state and inducing inflammatory cytokines and chemokines like IP-10 that recruit virus-specific T cells to the liver.

Despite the rapid viral sensing and induction of antiviral responses, HCV is able to persist as it has devised several mechanisms to evade recognition and inhibit IFN-signaling pathways. NS3/4A was shown to use its serine protease activity to act as the viral “Swiss army knife” to disrupt viral sensing pathways at early steps by splicing MAVS and TRIF downstream of the major sensors of HCV–RNA [reviewed in Ref. (70)]. Other HCV proteins as core, E2, and NS5A interfere with various early and late steps along the JAK–STAT signaling pathway that is induced by type I and III IFNs further contributing to inhibiting the IFN response (88–90). However, ISGs remain highly expressed in individuals who develop chronic infection and their level of expression correlates inversely with the response to IFN based therapies (91). This suggests that sustained viral replication and induction of ISGs induce a state of tolerance within hepatocytes that become refractory to external IFN stimulation.

### Natural killer cells

Natural killer cells are one of the earliest lines of innate immune defense. They exert their function by killing virally infected cells via secretion of cytotoxic molecules like granzymes and perforin or through tumor necrosis factor (TNF)-related apoptosis-inducing ligand (TRAIL)-mediated killing. They also secrete cytokines that can regulate innate and adaptive immunity like IFNγ, TNFα, IL-10, and IL-21. NK activity is governed by the balance between activating and inhibitory signals. One of the major determinants of such activity is the strength of the interaction between the inhibitory KIRs expressed on NK cells and their MHC class I ligands expressed on target cells (92). NK cells are highly enriched in the liver and are expected to play a major role in controlling hepatotropic infections (93, 94). NK cells were activated early in healthcare workers following accidental percutaneous exposure to HCV and may have contributed to protection from infection in 11/12 individuals in this group who remained aviremic (95). NK cells were also activated in high risk IDUs and expression of the NK cells activating receptor NKp30 correlated with protection from HCV infection (96). Hyperactivation of NK cells was observed during acute HCV infection irrespective of the outcome (97–99). Increased IFNγ production was enriched in KIR2DL3 expressing NK cells as suggested by genetic studies demonstrating that this allele was associated with spontaneous resolution of HCV potentially due to less NK cell inhibition (98). Reduced expression of NKp30 could predict the outcome of acute HCV (97). Notably, NK cells from HCV acutely infected individuals and during chronic infection were biased toward cytotoxicity rather than cytokine production (99, 100) and NK cell degranulation, a surrogate marker of cytotoxicity, correlated with the magnitude of HCV-specific T cells (99). This suggests that NK cell cytotoxic activity may kill infected hepatocytes and facilitate the transfer of HCV antigens to the draining lymph nodes as they are picked up and presented by DCs.

During chronic HCV infection, intrahepatic NK cells are generally highly activated and this activation correlates with the degree of liver inflammation (101, 102). Peripheral NK cells express variable levels of the NK activation markers NKp30, NKp44, NKp46, NKG2C, and NKG2D and increased expression of the inhibitory receptor NKG2A as compared to healthy individuals [reviewed in Ref. (103–105)]. NK cell numbers and functions, especially cytotoxic activity, were shown to be lower in chronic hepatitis C patients as compared to healthy donors but results varied from one study to another probably reflecting various experimental designs and patient cohorts (106–109). Recombinant HCV E2 protein was reported to directly bind CD81 on the surface of NK cells and inhibit its functions (110, 111). HCV-infected hepatocyte cell lines but not purified virions reproduced a similar phenomenon (112, 113).

Recent data from the lymphocytic choriomeningitis virus (LCMV) model has demonstrated that NK cells may act as rheostat to control CD4-mediated help for CD8 T cell functions during chronic infection (114). Similarly, in the hepatitis B virus (HBV) model, it was shown that NK cells can selectively eliminate activated T cells expressing the TNF-related apoptosis-inducing ligand death receptor 2 (TRAIL-R2) and thus, negatively regulate antiviral immunity (115). NK cells also interact with DCs and this cognate interaction regulates both innate and adaptive immunity. NK cells produce IFNγ and TNFα that induce maturation of DCs and enhance their capacity to prime virus-specific T cells. In return, DCs produce IL-12 and IL-15 that enhance activation of NK cells (116). It would be interesting to examine if such cross talk exists during acute HCV infection and cross-regulates adaptive immunity.

### Dendritic cells

Dendritic cells are one of the major antigen-presenting cells (APCs) in the body. They bridge innate and adaptive immunity and may impact priming of HCV-specific immune responses. DCs rapidly differentiate into mature DCs in response to various “danger” signals like activation through pathogen associated molecular patterns (PAMPs) in particular TLR ligands, interaction with innate lymphocytes (NK and NKT cells), cytokines, and inflammatory mediators (117). There are two main subsets of DCs, mDCs representing the majority of DCs and mostly associated with antigen processing and presentation and pDCs that can sense viral infections and are the main producers of type I and type III IFNs. pDCs can detect HCV RNA in a TLR-7 specific manner when presented as part of an infected cell (86). This activation can be mediated by transfer of exosomes containing HCV–RNA from infected cells to pDCs (87). As such, DCs are considered a main orchestrator of the HCV innate and adaptive immune response.

The role and function of DCs during acute and chronic HCV infection remain highly controversial. The frequencies of mDCs and pDCs were shown to correlate with the outcome of infection, where reduced frequencies were associated with chronic infection (118–121). Our group has recently demonstrated that sustained hyperresponsiveness of DCs was associated with resolution of HCV infection suggesting better priming of HCV-specific T cells (122). Various groups have reported that DCs are defective in chronic HCV, in particular, in response to TLR ligands (123–126), may be infected (123–126), and may induce proliferation of Tregs (127) while others have demonstrated that they are functional (118, 120, 128, 129). How do DCs influence priming of HCV-specific immune responses? Is it through a direct effect on antigen presentation or through better NK–DC interaction? Are DCs presenting HCV antigens more tolerogenic or targets for elimination by NK cells? These remain as some of the gray areas in the HCV field.

## Adaptive Immune Responses during Acute Primary HCV

The importance of cell-mediated immunity in HCV clearance is demonstrated by the correlation between specific HLA class I and class II alleles and spontaneous resolution and underscored by depletion studies in the chimpanzee model showing that both CD4 and CD8 T cells are required for viral clearance and that they have complementary rather than exclusive roles. The role of nAbs is slowly being unraveled. In the following sections, we will summarize the current state of knowledge on this topic.

### Onset of adaptive immune responses

HCV-specific CD8 and CD4 T cell responses appear late around 6–8 weeks following primary HCV infection despite high levels of viral replication. This delay in the onset of virus-specific T cells could be attributed to the tolerogenic nature of the liver environment and the time required for viral antigens to reach the draining lymph nodes to be presented by professional APCs (38, 94). It might also be indicative of the capacity of the virus to evade the innate immune system, interfere with maturation of DCs or impair their capacity for antigen processing and presentation. Onset of HCV-specific CD8 T cells in blood and/or liver and the detection of IFNγ, CD3, CD4, and CD8 transcripts in the liver are kinetically linked to decline in viral loads (76, 77).

### CD8 T cell responses during acute primary HCV

The breadth of the CD8 T cell response is a key determinant of spontaneous resolution. Up to nine different epitopes were recognized simultaneously in chimpanzees and humans with acute resolving HCV while much fewer epitopes were recognized in individuals developing chronic infection (130, 131). Responses targeting the NS proteins were immunodominant and correlated with spontaneous clearance (132), but the individual epitopes targeted were different, even among individuals with shared HLA alleles (133, 134).

Escape mutations within targeted CD8 T cell epitopes are associated with viral persistence and represent a major immune evasion mechanism used by the virus. They typically occur early and become fixed in the viral quasispecies (135–137). Mutated epitopes are mostly associated with loss of binding to the restricting MHC and are thus not recognized or poorly induce a new T cell response (135, 136). Escape mutations are also dependent on the interplay between the virus and host genetics. The host HLA alleles enforce selective pressure on their cognate epitopes. This is evident as these mutations revert to their wild type sequences when transmitted to an individual not carrying the same HLA allele and where the epitope is no longer under selection pressure (138). Host HLA may also induce selection pressure on a population level resulting in viral adaptation within a specific genetically related population (139). Finally, viral fitness limits the variability within some epitopes (140, 141). Certain HLA-alleles like HLA*B27 are considered protective as they prime responses to highly constrained epitopes that are less likely to mutate because of the high fitness cost (65, 142, 143).

The epitope-specific CD8 T cell receptor (TCR) repertoire may also limit escape mutations. One study in the chimpanzee model suggested that generation and maintenance of a repertoire with higher diversity was associated with resolution of HCV infection and limited the emergence of escape mutations in the targeted epitopes (144). Another study correlated a specific mutation in an MHC class I restricted epitope (NS3_1406_) to the scarcity of specific TCRs that could recognize the mutant, thus exploiting this “hole” in the T cell repertoire (67). Several studies in HIV cohorts demonstrated an association between the ability to control HIV replication and progressing to AIDS with the presence of specific CD8 T cell clonotypes that possessed superior functional avidity, as well as cross-reactivity to the different variants of the targeted epitope (145). Additional studies examining the dynamics of the TCR repertoire, functionality, and escape mutations during acute and chronic HCV in relation to changes in viral quasispecies would be of interest.

The magnitude of responding CD8 T cells also correlates with spontaneous resolution. Studies using MHC class I tetramers demonstrated that T cells specific for one single epitope can reach up to 8% of CD8 T cells in spontaneous resolvers (131, 146). The frequency of HCV-tetramer positive population was several folds higher in the liver than in peripheral blood of chimpanzees during acute resolving HCV with elevated expression of CD69, a marker of T cell activation (147).

The use of MHC class I tetramers allowed direct *ex vivo* phenotypic characterization of the HCV-specific CD8 T cells. Early expression of the IL-7 receptor alpha (CD127) on virus-specific CD8 T cells emerged as a major predictor of spontaneous resolution while its loss was associated with viral persistence (148, 149). CD8 memory T cell populations generated following clearance of primary HCV infection were CD127^hi^ and Bcl-2^hi^ (146). This is consistent with data from the LCMV model showing that CD127 is a marker of cells destined to become long-lived memory T cells (150). On the other hand, HCV-specific CD8 T cells express variable levels of PD-1 during acute infection suggesting that PD-1 acts more as an activation rather than an exhaustion marker (151–153). Other exhaustion markers like the T cell immunoglobulin and mucin domain 3 (Tim-3), cytotoxic T lymphocyte associated antigen-4 (CTLA-4), CD160, KLRG-1, and 2B4 are differentially expressed during acute and chronic HCV suggesting a spectrum of exhaustion that correlated with function and persistent infection (154, 155). The Tim-3 ligand galectin-9 (Gal-9) was upregulated in the plasma during acute infections progressing to chronicity (155, 156) and in the liver of chronic individuals (156), thus contributing to T cell exhaustion. Blockade of PD-1, Tim-3, and CTLA-4 separately or in combination rescued HCV-specific CD8 T cells from exhaustion suggesting that they act synergistically (157–159). *In vitro* supplementation of IL-21, a mediator of CD4 T cell help, also rescued HCV-specific T cells from Tim-3/Gal-9-mediated apoptosis (155).

HCV-specific CD8 T cells become difficult to detect in the peripheral blood of individual who develop chronic infection but are readily detectable in the liver and continue to express a highly activated and exhausted phenotype (160, 161). HCV-specific intrahepatic CD8 T cells from chronic HCV patients expressed TIM-3, PD-1, and 2B4, while CD8 T cells from patients who had cleared the virus following IFN therapy (i.e., in absence of active viral replication) and T cells specific for cytomegalovirus lacked TIM-3 and expressed higher levels of LAG-3; these cells also exhibited different memory phenotypes and proliferative responses (162).

HCV-specific T cells in the peripheral blood might be slightly impaired in their proliferative and cytokine producing capacity when they first appear in blood suggesting a “stunned” phenotype (163). Analysis of multiple effector functions simultaneously identified the presence of a polyfunctional population of virus-specific CD8 T cells that correlated with spontaneous clearance of HCV (146), similar to what was observed during control of HIV infection (164) and in the LCMV model (165). The functions analyzed included secretion of the effector cytokines IFNγ, the T cell growth factor IL-2 and the degranulation marker CD107a, a surrogate marker of cytotoxicity (146). Assays on sorted cells demonstrated that polyfunctionality was localized within the CD127^+^ HCV-tetramer reactive CD8 T cells providing further evidence to the importance of this T cell subset in mediating viral clearance (146).

Differences in CD8 T cell functions become increasingly apparent as the infection progresses and are associated with loss of helper CD4 response, as well (discussed below). In patients who become chronically infected T cells show progressive loss of function, reduced polyfunctionality and diminished proliferative capacity (146, 166). CD8 T cells detected in chronic HCV patients were arrested in an early maturation stage, impaired in cytokine production, cytotoxicity, and/or proliferative capacity (167–169). The loss in function also correlates with the degree of exhaustion as described above and were reversible *in vitro* upon blockade of the PD-1, CTLA-4, and/or Tim-3 pathways (170). However, *in vivo* blockade of the PD-1 pathway in chronically infected humans and chimpanzees had limited efficacy suggesting that a threshold of functional HCV-specific T cells is required for such immunotherapeutic strategies to work (171, 172). Functional exhaustion could be attributed to the persistence of the antigen, where it was shown in the LCMV model that prolonged exposure to viral antigens is the main cause for reduced frequency and impaired effector functions of virus-specific CD8 T cells (173, 174). Confirming this hypothesis are data demonstrating that HCV-specific CD8 T cells detected in chronic patients where the respective cognate epitope was mutated (i.e., no longer stimulated by the cognate epitope) were functional and expressed lower levels of PD-1 and increased CD127, a marker of long-lived memory cells and a predictor of spontaneous resolution (175–177). Similarly, early therapeutic intervention and sustained virologic response to IFN therapy rescued CD127^+^ long-lived memory T cells (146, 176).

In summary, spontaneous resolution of acute HCV correlates with early emergence of CD127^+^ HCV-specific CD8 T cells. The response is also broad, of high frequency and polyfunctional in nature. Several factors contribute to failure of this response in individuals who become persistently infected. First, escape mutations in targeted epitopes facilitate viral evasion of the immune system. Second, loss of CD4 help or a switch to an immunoregulatory profile further compromises the antiviral capacity of virus-specific CD8 T cells (discussed below). Third, continued viral replication contributes to exhaustion of HCV-specific T cells through persistent antigenic stimulation leading to progressive loss of function and diminished survival of virus-specific CD8 T cells. Fourth, exhaustion is exacerbated by increased expression of the ligands to the inhibitory receptors like Gal-9.

### CD4 T cell responses during acute primary HCV

The importance of CD4 T cells in clearance of primary acute HCV was first demonstrated by the correlation between CD4 proliferative responses targeting the HCV NS proteins and viral clearance (178, 179), whereas the loss of such responses was associated with viral recurrence (37, 131, 180). The breadth and vigor of the CD4 response also correlated with spontaneous resolution (179, 180). CD4 T cell responses targeting up to 14 different epitopes were detected in individuals with acute resolving HCV (181) and some of these epitopes were identified as promiscuous epitopes that can be presented by multiple HLA-DR alleles (182, 183). Unlike CD8 epitopes, escape mutations were uncommon in MHC class II restricted CD4 T cell epitopes suggesting that this is an unlikely mechanism of CD4 T cell failure (184, 185).

Despite the development of MHC class II tetramers and multimers, direct *ex vivo* detection and characterization of HCV-specific CD4 T cells has been limited due to the low frequency of tetramer reactive T cells, very often requiring enrichment. In addition, the restricted number of epitopes and tetramers that are available limited such analysis. Direct *ex vivo* studies have demonstrated that HCV-specific CD4 T cells are detectable by tetramers mostly during the acute phase. The frequency declines and they remain detectable in humans who resolve the infection during the memory phase but require enrichment where they were shown to express markers of central memory T cells (CCR7^+^CD45RA^−^CD27^+^) (186, 187). Nevertheless, HCV-specific CD4 T cells were detectable directly *ex vivo* by MHC class II tetramers in one chimpanzee at 7 years post resolution of acute primary HCV (152). The use of tetramers also revealed that broad virus-specific CD4 T cells were present early in most individuals acutely infected with HCV irrespective of the outcome, but rapidly lost the proliferative capacity and cytokine production, specifically IL2, and disappeared from the periphery with the establishment of chronic infection (188, 189). This challenged the prevailing hypothesis that HCV persistence is the result of a failure to prime virus-specific CD4 T cells and suggested that it is rather T cell exhaustion and the failure to sustain such CD4 T cell responses that leads to chronicity. Indeed, *in vitro* blockade of the PD-1, IL-10, and TGF-β pathways rescued the proliferative and cytokine producing capacity of CD4 T cells from individuals with chronic HCV (190).

The importance of CD4 T cell help in maintaining a functional CD8 T cell response during HCV infection was clearly demonstrated by CD4 depletion studies in chimpanzees, where the frequency of virus-specific CD8 T cells and their cytokine producing capacity gradually declined resulting in accumulation of escape mutations in the targeted CD8 epitopes (191). So how do HCV-specific T cells provide help for CD8 T cells? Our group recently demonstrated that expansion of CD161^hi^CCR6^+^CD26^+^CD4^+^ Th17 T cells correlated with spontaneous resolution of HCV. These cells produced large amounts of IL-17A and IL-21 and this correlated with the increased plasma concentration of these two cytokines and spontaneous resolution of HCV (155). Specifically, increased plasma levels of IL-21 during the late acute phase correlated with the frequency of virus-specific CD8 T cells and rescued them from Tim-3/Gal-9-mediated apoptosis (155). This underscored the role of IL-21 as a major helper cytokine similar to previous observations on the LCMV model and HIV (192). Other sources of IL-21 like NK cells and follicular helper T cells (Tfh) remain unstudied and may play an active role in mediating CD4 help.

Virus-specific Th17 and regulatory T cells (Tregs) were detected during acute and chronic HCV (193–195) and Th17-like CD4 T cells were enriched in the livers of individuals with chronic HCV (196). The preferential expansion of Tregs with viral persistence may represent another level of inhibition of HCV-specific CD4 and CD8 T cells. CD39^+^CTLA4^+^ Tregs were expanded during acute HCV infections progressing to chronicity with a shift in the Th17/Treg balance (155). Such Tregs can be a source of Gal-9 further contributing to the inhibition/exhaustion of CD4 and CD8 T cells expressing Tim-3 (155). Tregs are also expanded in chronic HCV but it is not yet clear if expansion of Tregs is a cause or an effect of HCV persistence as a feedback mechanism to limit virus induced immunopathology and inflammation. A recent report suggested that subinfectious exposures to HCV in the chimpanzee model may predispose to the development of Tregs that can later suppress HCV-specific responses upon subsequent infection (197) suggesting that elevated Tregs can be a cause of persistence. Finally, the flexibility and reversible nature among the different T-helper subsets is a domain of intense research (198) and whether HCV-specific CD4 T cells are endowed with such plasticity and the pathways involved remain to be seen.

In summary, broad HCV-specific CD4 T cell responses are primed during most acute HCV infections. However, contraction of this CD4 T cell population and its failure in sustaining a robust CD8 T cell effector response is a hallmark of HCV persistence. Early induction of IL-21 producing Th17 cells is critical to mediate help and limit exhaustion of virus-specific CD8 T cells. However, CD4 T cell exhaustion, leads to sequential loss of IL-2 production, proliferative capacity, and IFNγ production in addition to an imbalance in the Th17/Treg ratio. Tregs may contribute to exhaustion and inhibition of CD4 and CD8 T cells through production of the Tim-3 ligand Gal-9 and the regulatory cytokines IL-10 and TGF-β.

### Humoral responses during acute primary HCV

Although, HCV RNA reaches high serum titers by week 2 post infection, anti-HCV antibody response (seroconversion) is usually not detected before week 8 (199, 200). Early studies showed that antibodies (Abs) targeting the HVR-1 region of the E2 glycoprotein of HCV are neutralizing *in vitro* and *in vivo* (201, 202), but select for mutations in the envelope region (203). Chimpanzee studies showed that generation of Ab responses was not necessarily associated with viral clearance (204, 205). In humans, Ab responses were delayed, were of low titers and declined rapidly in individuals who cleared HCV spontaneously (206–208). One study showed that nAbs appeared in patients after HCV has already established chronic infection and were unable to clear the virus and selected for escape mutants (209). However, another study showed that early induction of nAbs was associated with spontaneous resolution of HCV primary infection (210). Broadly nAbs that could protect against heterologous HCV infection were recently reported (211, 212). Similar broadly nAbs confer protection against most pathogens, yet, they did not necessarily correlate with the control of infection or protection against superinfection in some chronic viral infections like HIV (213).

One of the major limitations for understanding humoral immunity against HCV is the lack of proper tools to measure precisely the levels of nAbs. The current method measures neutralization of HCVpp or infectious virions carrying HCV E1–E2 envelope glycoproteins corresponding to a limited number of HCV genotypes reference sequences, and thus do not necessarily represent the autologous E1/E2 sequences circulating in the patient (214, 215). An HCVpp library comprised of 19 genetically-distinct sequences that represent the natural variability of genotype 1 E1/E2 was recently used to demonstrate the evolution of the HVR-1 sequences in response to nAbs (216). A recent study using this HCVpp library demonstrated that resolution of HCV infection was associated with a broad nAb response generated early during the infection (217).

Another antiviral role for anti-HCV Abs could be through induction of antibody-dependent cellular cytotoxicity (ADCC). ADCC is a mechanism whereby the variable region of Abs binds to infected cells while the constant region (Fc) is recognized by Fc receptors expressed by various innate immune cells including NK cells. Binding to the FcγR3a (CD16) triggers cytokine production and degranulation of NK cells, resulting in lysis of the targeted cell. Several studies suggest that ADCC plays a role in controlling viral replication during HIV infection (218, 219) and may have contributed to the success of the HIV RV144 vaccine trial performed in Thailand (220). So far, only one study has demonstrated the presence of ADCC-inducing antibodies specific for the E2 envelop protein in several HCV patients during acute and chronic HCV infection, as well as after spontaneous clearance (221). Further studies will be required to confirm these results and determine if ADCC-inducing antibodies play a role in the spontaneous clearance of HCV infection and in the protection against HCV infection and reinfection.

Some major challenges exist for generating protective humoral immunity against HCV. First, the envelope proteins are not highly immunogenic, causing the Ab response to be slow and weak during primary infection (216). Second, Abs mostly target the HVR of E2, a region with high mutation rates, which facilitates the selection of viral sequences that are highly resistant to Ab neutralization (222). This selection was shown to take place as the cellular immunity collapsed and the infection progressed to chronicity (223). Third, the epitopes targeted by nAbs are shielded by heavy glycosylation and complexation with host lipoproteins, which limits their efficacy *in vivo* (224).

In summary, recent data suggest that nAbs are induced earlier than what was previously thought and may play an active role in spontaneous resolution but these results remain inconclusive and further investigation using autologous E1/E2 sequences is required to understand this role. The recent elucidation of the crystal structures of E2 (225) and E2 bound to the broadly nAb AR3C (226), as well as structural mutational studies should provide better insights into how E1/E2 interact with their receptors and nAbs. In addition, the role of CD4 T cells, in particular Tfh cells, in providing help for antibody production by B cells remains undefined.

## Summary of Acute Phase Immune Responses and Mechanisms of Immune Evasion

**Figure 2:**
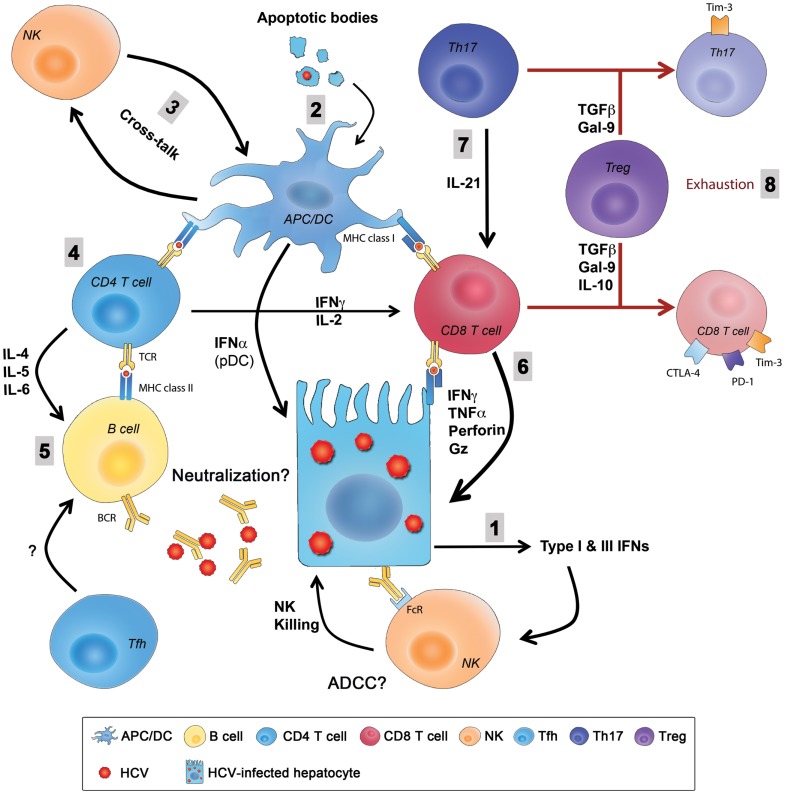
**Summary of innate and adaptive immunity during primary HCV infection**. (1) HCV entry and replication inside hepatocytes induces the production type I and type III IFNs that are also secreted by pDCs and create an antiviral state in infected hepatocytes and neighboring cells and stimulate NK cells that kill infected hepatocytes. (2) Liver resident and migratory APCs (Kupffer cells and DCs) uptake apoptotic bodies from destroyed HCV-infected hepatocytes and present HCV-derived epitopes to both CD4 and CD8 T cells in the context of MHC class II and MHC class I, respectively. (3) The cross-talk between DCs and NKs regulates the function of both cells enhancing antigen presentation and priming, as well as NK-mediated killing. (4) CD4 helper T cells support responses of CD8 T cells and B cells through production of Th1 and Th2 cytokine, respectively. (5) Whether Abs produced from B cells during primary infection have an essential role in viral clearance by directly neutralizing the virus or by mediating ADCC is not fully understood, and the role of Tfh cells in this process is still unknown. (6) CD8 T cells eliminate HCV-infected cells through direct cytotoxic mechanisms (cytolytic granules, as perforin and granzyme) or non-cytolytic mechanisms through secretion of the antiviral cytokines IFNγ and TNFα. (7) CD8 T cell functions are sustained and enhanced by IL-21 mainly produced by Th17 cells. IL-21 is also essential to rescue virus-specific T cells from exhaustion caused by persistent exposure to HCV antigens. Reduced IL-21 production or Th17 cell numbers results in an increased exhaustion status and expression of exhaustion markers like PD-1, Tim-3, CTLA-4, and others. (8) Regulatory T cells might be a cause in the failure of the primary immune response by secreting the regulatory cytokines TGFβ, IL-10, or secretion of Gal-9 that enhances apoptosis of Tim-3^+^ CD4 and CD8 T cells. ADCC, antibody-dependent cellular cytotoxicity; APC, antigen-presenting cell; CTLA-4, cytotoxic T lymphocyte antigen-4; DC, dendritic cell; FcR, receptor for the constant fragment of the antibody; Gal-9, galactin-9; Gz, granzyme; HCV, hepatitis C virus; IFN, interferon; IL, interleukin; NK, natural killer; PD-1, Programed death-1; pDC, plasmacytoid DC; Tfh, T follicular helper cells; TGF, transforming growth factor; Th17, T-helper 17 cell; Tim-3, T cell immunoglobulin and mucin domain 3; TNF, tumor necrosis factor; Treg, regulatory T cell.

Hepatitis C virus infection of hepatocytes activates innate immune sensing mechanisms like TLRs, RIG-I, and PKR that activate signaling cascades resulting in production of type I and type III IFNs and induction of several antiviral ISGs in infected hepatocytes. HCV can also be transferred to DCs or Kupffer cells via exosomes leading to activation of innate pathways and additional secretion of IFNs. Intrahepatic NK cells are activated by IFNs and recognize and kill HCV-infected hepatocytes generating apoptotic bodies containing HCV antigens. These apoptotic bodies are taken up by APCs like Kupffer cells and DCs that process and present HCV antigens as peptides bound to MHC class I and II and prime HCV-specific CD8 and CD4 responses, respectively. Infected hepatocytes also present processed HCV antigens in the context of MHC class I and prime virus-specific CD8 T cells that contribute to killing and generation of additional apoptotic bodies containing viral antigens. The transfer of HCV antigens from the liver to draining lymph nodes via migratory DCs is thought to be critical for priming of efficient immune responses. Several populations of HCV-specific CD4 helper T cells are primed including Th1 cells that provide help for CD8-mediated killing of infected hepatocytes via production of IFNγ and TNFα, Th17 cells that produce IL-21 that limits exhaustion of HCV-specific CD8 T cells and rescues them from apoptosis and Th2 cells that provide help for antibody producing B cells and generation of nAbs via IL-4 and IL-6. The role of Tfh cells and their support for development of nAbs remain undefined during acute HCV, as well as the potential contribution of ADCC to viral control. Similarly, cross-talk between NK cells and DCs and how this interaction influences priming of virus-specific T cells and regulates their function are unknown. HCV can evade innate immunity by interfering with the IFN-signaling pathway, inhibiting NK cells’ functions (even transiently), escape mutations of targeted CD8 T cell epitopes, or exhaustion of virus-specific CD4 and CD8 T cells through upregulation of exhaustion molecules like PD-1, Tim-3, CTLA-4, and others. Tregs are also induced and they can dampen HCV-specific responses directly through secretion of the immunoregulatory cytokines IL-10 and TGF-β or production of Gal-9 that can induce apoptosis of Tim-3^+^ virus-specific Th1, Th17, and CD8 T cells (Figure 2).

## Protective Immunity Against HCV

HCV-specific memory CD4 and CD8 T cells were detectable in the peripheral blood of humans who have spontaneously resolved primary acute HCV for up to 20 years following viral clearance while antibody responses waned (227). Similarly, memory CD4 and CD8 T cells were detectable in the peripheral blood and liver of HCV-resolved chimpanzees at 7 years following resolution of primary infection (147). Virus-specific CD4 and CD8 T cells were also detectable in individuals with potential occupational, household contact, or high risk behavior like injection drug use, in absence of detectable antibody responses or history of infection suggesting subclinical exposure, and priming of HCV-specific memory T cells (228–231).

The frequency of HCV-specific memory T cells was stable for several years after the resolution of primary infection in chimpanzees (147). Rechallenge studies demonstrated no sterilizing immunity but were characterized by a shorter duration of viremia and lower viral loads and were associated with protection from virus persistence in most (147, 232–235), but not all studies (236). Expansion of HCV-specific memory CD4 and CD8 T cells in peripheral blood and the liver was associated with rapid control of HCV infection upon rechallenge. IFNγ producing CD4 and CD8 T cells were detected at a higher frequency and much earlier during secondary infection as compared to primary infection in the same chimpanzees (147, 191, 232–235). The hierarchy of CD4 helper T cell epitopes was preserved during secondary infection years later (182). Control of viral replication in animals challenged with HCV following experimental vaccination was associated with priming of CD127^+^ PD-1^lo^ CD8^+^ T cells that persisted at high levels for prolonged periods and were bifunctional, producing both IFNγ and TNFα (237). Another study demonstrated that clearance of HCV reinfection upon heterologous rechallenge involved the activation of both innate and adaptive immune responses. Higher intrahepatic gene expression of CD56, CD8, type I and II IFN, and ISGs were observed in one out of two chimpanzees who were able to clear the rechallenge virus (238). This response correlated with expansion of HCV-specific CD4 and CD8 T cells in the peripheral blood in absence of nAbs (238).

The protective role of memory CD4 and CD8 T cells was confirmed using antibody-mediated depletion. CD4 T cell depletion was associated with low level viremia. Although memory CD8 T cells initially provided some control for viral replication, they rapidly waned in absence of CD4 T cell help leading to accumulation of escape mutations in targeted epitopes and viral persistence after more than 1 year of follow-up (191). In contrast, memory CD8 T cell depletion led to a significant delay in control of viremia, and this control coincided with the recovery of HCV-specific T cells in blood and liver (147). Altogether, these data demonstrate a protective role for memory T cells upon reexposure to HCV but the role of nAbs was not sufficiently analyzed due to lack of the proper tools and the generally weak anti-HCV antibody response induced in chimpanzees.

As described above, HCV-specific memory CD4 and CD8 T were detectable 20 years post-clearance of the virus, whereas antibodies were barely detectable (227). Phenotypic characterization of HCV-specific CD4 T cells in spontaneously resolved cases showed they were CCR7^+^, CD45RA^−^, and CD27^+^, characteristic of central memory T cells (186). Spontaneous resolution from a primary HCV infection was associated with the generation of polyfunctional HCV-specific memory population expressing CD127^hi^ Bcl-2^hi^ profile (146, 148, 149) consistent with a profile of long-lived memory CD8 T cells (150). It is not clear how HCV-specific memory CD4 and CD8 T cells are maintained for prolonged periods. Apart from their intrinsic long-lived properties and transcriptional profile, memory T cells can be maintained through homeostatic proliferation in response to the γ-chain cytokines IL-7, IL-15, and IL-21 (239, 240). They may also be maintained through periodic restimulation with cross-reactive viral or self-antigens. Finally, it is possible that minor quantities of HCV RNA are maintained for a prolonged period following viral clearance and contribute to periodic restimulation of such memory T cells (241, 242).

The protective role of HCV-specific immunological memory is difficult to address in humans. Nevertheless, epidemiological studies demonstrated that high risk IDUs who have spontaneously resolved one HCV infection were less likely to be reinfected than HCV-naïve individuals despite repeated high risk exposures (243–246), but the reported rate of incidence of reinfections was variable because of the different HCV RNA testing interval where reinfections of shorter duration or accelerated viral clearance may have been missed (247). Other factors include differences in the characteristics of individual subjects, as age, ethnicity, and risk behaviors (247). Nevertheless, the rate of spontaneous clearance of reinfection was higher than that for primary infection (243, 245, 248). This provided preliminary evidence of protective immunity against HCV. Osburn et al. monitored a group of high risk IDUs at monthly intervals and observed a rate of reinfection at almost 50% (11/22). Spontaneous clearance of reinfection was associated with broadened T cell responses and generation of cross-reactive Ab responses (248). However, phenotypic and polyfunctional characterization of HCV-specific memory CD4 and CD8 T cells was not performed. In this study, 10/11 subjects examined resolved the second infection, thus a control group of unprotected patients that would enable the definition of the exact correlates of protection was lacking. Preliminary work from our group suggests that protection from HCV persistence upon reinfection is variable and associated with expansion of HCV-specific memory CD8 T cells that are polyfunctional in nature with expansion of a CD127^lo^ population consistent with effector memory T cells (249).

Early treatment of HCV could rescue long-lived HCV-specific memory CD4 and CD8 T cells (146, 176, 189) but the protective role of natural T cell memory generated after viral clearance versus treatment induced memory generated after therapeutic-mediated clearance remains to be evaluated. An equally protective memory immune response would argue in favor of early treatment of high risk individuals as this will provide them with long-term protection if they are reexposed. Comparison of the capacity of the new DAAs and/or IFN free regimens to rescue such protective memory responses should also be evaluated.

**Figure 3:**
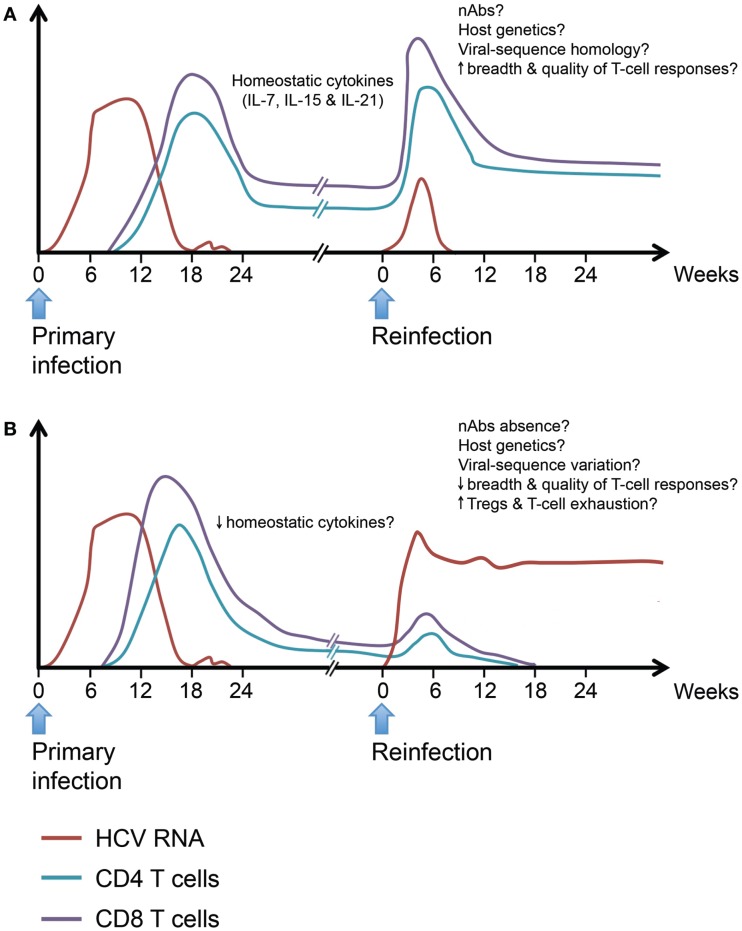
**Hypothetical model for protective and non-protective immunity upon HCV reinfection**. Individuals who spontaneously clear primary HCV infection develop long-lived virus-specific memory CD4 and CD8 T cells. Such memory T cell populations are maintained by the action of homeostatic cytokines like IL-7, IL-15, and IL-21. Upon HCV reinfection, some individuals will be protected against viral persistence while others will be unprotected. **(A)** The protected individuals will be able to spontaneously clear the second infection with a shorter period of viremia and reduced peak viremia. Viral clearance will be accompanied by an accelerated memory T cell recall response detectable in both liver and blood. The exact characteristics of a protective response are yet to be defined in terms of breadth and quality. **(B)** In unprotected individuals, reinfection will be associated with a weak or late recall response and incomplete T cell-mediated control of viremia. The underlying causes of failure to resolve the reinfection despite the ability to spontaneously resolve a prior infection maybe low levels of homeostatic cytokines affecting the maintenance of memory T populations, reduced breadth, or quality of the recall response upon reinfection and increased frequency of Tregs leading to dampening of the immune response, viral persistence, and rapid exhaustion of HCV-specific T cells. Neutralizing antibodies, host genetics, and homology between the infecting viral sequences at different episodes may play a role in protection upon reinfection.

In summary, long-lived T cell memory responses are generated following spontaneous HCV clearance and data from chimpanzees and limited human studies suggest that they can provide some protection. However, the signatures of a protective immune response upon reexposure are far less defined, especially in terms of the phenotype and functionality of HCV-specific protective memory immune responses. We propose a model inferred from our current knowledge of protective immunity during acute primary HCV infection (Figure 3). We hypothesize that protection upon reexposure is associated with the maintenance of higher frequency, breadth, and polyfunctionality of HCV-specific memory CD4 and CD8 T cells. Such long-lived memory T cells are likely maintained through homeostatic proliferation in response to γ-chain cytokines. Upon reexposure, these memory T cells expand rapidly and eliminate the infecting virus. Similar to murine models of viral infections, repeated exposure will select for the virus-specific memory T cells with the highest proliferative capacity and functional avidity (250). It is likely that such long-lived memory T cells will acquire a genomic signature that will facilitate their capacity to expand rapidly and effectively upon reexposure. Individuals who cannot maintain such long-lived, broad, and polyfunctional memory T cells because of reduced homeostatic cytokine or proliferation or predisposition to generation of Tregs and increased T cell exhaustion are less likely to be protected upon reexposure. Host genetics, nAbs and homology between the infecting viral strains during primary and secondary infection can tip the balance toward viral clearance or persistence of the second infection.

Protective immunity in the context of real life exposure remains the most understudied area of HCV due to the limited number of organized high risk cohorts suitable for such studies and the need for follow-up at close intervals to detect multiple infection episodes (247). Concerted efforts to study such response on a large scale in matched cohorts with different ethnic backgrounds are underway and should yield important knowledge in the future (251, 252). In addition, the organization and characterization of such cohorts provides the perfect setup to evaluate vaccine candidates. Use of novel genomic and proteomic tools should yield better insights about the pathways involved in protective immunity. Comparative analysis of virus-specific cellular and humoral responses in relation to variations in the infecting viral strain is essential to understand the interplay between the virus and immune system and cross-protective immunity.

## Implications for Vaccine Development

The need of a prophylactic HCV vaccine to prevent viral transmission remains an urgent need to reduce the future disease burden worldwide (11). The plethora of data suggesting that cellular immunity is the major arm mediating spontaneous clearance during primary HCV infection led to the assumption that it should be the target for vaccine development. Nevertheless, the recent data on the role of nAbs in resolution of primary infection suggest a possible contribution of humoral immunity in both primary HCV clearance and prophylaxis.

There are two challenges facing the development of HCV vaccines. First, the genetic diversity of HCV sequences and its high rate of mutability. Second, the ability of HCV proteins to disrupt and evade the different arms of the immune system. This necessitates the design of an effective vaccine that generates memory immune cells capable of mounting a prompt recall response upon reexposure to diverse HCV strains in a fashion that outpaces the virus and prohibits it from crippling the immune system.

Three major approaches have been adopted for vaccine design against HCV. The traditional approach uses recombinant envelope proteins to induce nAbs. It has gained more potential recently with the discovery of broadly nAbs. Vaccination with recombinant E1/E2 proteins was shown to induce cross-reactive nAbs that might contribute to protective immunity upon exposure to HCV (253–255). The second approach uses virus-like particles (VLPs) that express HCV structural proteins to induce both humoral and cellular immunity, but it failed to induce humoral responses when tested in chimpanzees. Furthermore, upon homologous rechallenge 2/4 vaccinated chimpanzees demonstrated sporadic very low levels of viremia during 1 year of follow-up (256). The third and most promising approach is designing an HCV vaccine that would induce a potent T cell immune response. Replication-defective recombinant viral vectors [e.g., adenovirus (Ad), vaccinia virus (VV), modified vaccinia Ankara (MVA)] were used to deliver HCV antigens to prime T cell responses. Another promising vector that was successful in the simian immunodeficiency virus (SIV) model, but has not yet been tested in HCV is cytomegalovirus (257). HCV proteins could also be delivered using DNA vaccines, where recombinant plasmids expressing various proteins could be injected into the host [reviewed in Ref. (11)].

A combination of viral vector prime and DNA or recombinant protein boost is preferred to prevent neutralization of the subsequent boosts by vector-specific antibodies that could have been generated during priming. Two candidate vaccines using this strategy showed promising preliminary results and are at an advanced stage of development. The first vaccine uses heterologous prime/boost regimens with chimpanzee adenovirus Ad3Ch3 and a rare strain of human adenovirus (Ad6) expressing the entire NS region of genotype 1b BK strain (NS3-5B). This vaccine was tested in a phase I clinical trial in humans (ClinicalTrials.gov NCT01436357). Both vectors primed broad CD4 and CD8 T cell responses that were capable of responding to heterologous strains of HCV genotypes 1a and 3a. Polyfunctional HCV-specific T cells (IL-2^+^ IFNγ^+^ TNFα^+^) could be sustained for at least a year after boosting with the heterologous vector (258). This vaccine is currently in a phase II trial in high risk IDUs. The second vaccine uses a regimen of priming with Ad6 encoding NS3-5B of genotype 1b BK strain and boosting with NS3-5B-encoding plasmid DNA. A study in chimpanzees that received this vaccine then challenged with HCV demonstrated that control upon HCV challenge following vaccination was associated with CD127^+^ PD-1^lo^ CD8 T cells that persisted at high levels for prolonged period and were bifunctional (IFNγ^+^ TNFα^+^). Nevertheless, three out of five chimpanzees were not protected suggesting that a more efficient immune response may be required (237, 259).

Correlates of protective immunity against HCV need to be clearly defined so that they could be monitored following vaccination to predict the degree of protection that may be conferred. This should involve characterization of the genomic and proteomic pathways involved and identification of biomarkers that can be used as benchmarks to monitor vaccine success in a non-invasive way. Strategies targeting both cellular and humoral immunity are likely to be more successful (260). The use of immune modulators like PD-1 blockers, TLR agonists, or modulators of biological pathways like transcription factors involved in memory T cell generation may be useful as vaccine adjuvants to enhance immunogenicity and favor the development of the desirable immune response (250).

## Future Research Directions

There is a general misconception that this is the end of HCV and that the new treatments will completely eliminate HCV within the next few years. Unfortunately, this is highly unlikely since the new DAAs remain expensive and are associated with a fair number of side effects. Moreover, their use in special populations like individuals with advanced liver disease or HIV coinfection remains experimental and limited at this point. Most importantly, many of the individuals infected with HCV have not been tested and continue to infect others. Future research should focus on the many shades of gray that still cloud our understanding of what constitutes a long-term protective immune response and the interaction between the virus and the host. This requires the use of organized and well characterized cohorts and standardized methods to evaluate immunity on a large scale with sufficient statistical power. Areas of interest involve better understanding of the role of genetic predictors of HCV outcome like IL28B and IFNL4 in viral clearance and long-term protection; understanding the nature of CD4 T cell help for both CD8 T cells and antibody producing B cells; elucidation of the role of neutralizing and non-nAbs during primary and secondary HCV infections; development of better small animal models for preclinical testing of vaccines and the use of novel genomics and proteomics tools to identify implicated pathways and non-invasive markers of efficient immune response. Collaboration with other fields, including research on vaccines for other viruses like yellow fever virus and HIV, as well as work with more physiologically relevant *in vitro* models like primary hepatocytes should provide a clearer image of the protective immunity against HCV and better success for ongoing and future vaccine development strategies.

## Conflict of Interest Statement

The authors declare that the research was conducted in the absence of any commercial or financial relationships that could be construed as a potential conflict of interest.
